# Influence of Cost of Care and Adherence in Glaucoma Management: An Update

**DOI:** 10.1155/2020/5901537

**Published:** 2020-04-08

**Authors:** Frances Meier-Gibbons, Marc Töteberg-Harms

**Affiliations:** ^1^Eye Center Rapperswil, Merkurstr. 50, Rapperswil-Jona 8640, Switzerland; ^2^UniversityHospital Zurich, Department of Ophthalmology, Frauenklinikstr. 24, Zurich 8091, Switzerland

## Abstract

The costs for glaucoma care are rising worldwide. The main reason is the increase of life expectancy and the increasing variety of diagnostic tests and therapeutically options by implants and devices. How can we influence the increase in costs? Does a relationship exist between the rising costs and the behavior of patients especially in regard to adherence of patients to the prescribed therapy? Are there ways to improve adherence? The costs of a disease can be estimated by adding the direct costs and the indirect costs deriving from the disease. Many studies have been looking at the direct costs, for example, the costs of diagnostic tests and treatment modalities. Unfortunately, not many studies investigated the indirect costs, i.e., costs related to the need of a person to accompany the patient during his or her outpatient visits or the costs deriving from loss of work capacity because of the disease itself or the outpatient visits. Adherence or the synonym compliance has been discussed since many years, and it seems that it remains a major problem in the management of many chronic diseases. Despite all efforts to improve adherence, the adherence rate in chronic diseases such as glaucoma or arterial hypertension remains considerably low. One of the main factors in improving adherence is raising patient's awareness of the disease by providing general understanding of their disease. Other important factors are simplified therapeutic regimens, e.g., fixed combination drops, sustained drug release techniques, or new glaucoma surgical procedures with a more favorable risk profile.

## 1. Introduction

Glaucoma is one of the major causes for irreversible blindness worldwide. Quigley [1] estimated in 2006 that, by 2020, 80 million people will be affected. Tham [2] in 2014 mentioned that the number of patients with glaucoma will be 111.8 million by 2040. Glaucoma in early stages is mainly asymptomatic and thus, many patients are not aware of their disease. In the developed world, about half of the patients do not know that they suffer from glaucoma.

The prevalence of glaucoma is rising in a nonlinear fashion with age. The number of elderly people is rising and therefore, more patients will suffer from glaucoma in the future. The increasing digitalization and education of the population plays an important role. Many patients over the age of 65 work on a computer and drive their cars nowadays. On the other hand, the new technical possibilities for detecting a disease and the therapeutic options to treat it lead to an increasing demand from patients. Having the possibilities of detecting and treating the disease is one part of glaucoma management, and the other part is the behavior of the patients. Glaucoma, as many chronic diseases, has a low adherence and persistence rate which may lead to a progression of the disease and hence, an increase of costs [3]. In an earlier review [4], we studied the possible connections between adherence and costs of glaucoma care and concluded that improving adherence could reduce the costs of the disease by reducing the progression of the disease. The WHO has shown that the average healthcare costs have increased worldwide since 2009 despite a decrease in the real growth rates per capita, which is partially influenced by the economic crisis [5]. All the factors mentioned above influence the rising healthcare costs around the world. The question arises, if this vicious cycle can be interrupted or slowed down.

## 2. Materials and Methods

A literature search was performed using “PubMed,” search strings were “adherence” and “glaucoma” or “costs of glaucoma care.” Only publications in English, published until Dec 31, 2019, were included.

## 3. Results

The results were divided into (1) costs of glaucoma care, (2) patients adherence, and (3) improvement of adherence. (4) The last paragraph discussed the question whether a connection between costs and adherence exists.

### 3.1. Costs of Glaucoma Care

Schmier [6] mentioned in 2007 that studies published on costs in glaucoma care focus mainly on direct costs, i.e., costs of diagnostic tests and therapies (drugs, surgery, or laser) and the costs for transportation of the patient to his or her visits. The indirect costs, which are as important as direct costs, however, are seldom looked at in studies. The indirect costs include the costs for the accompanying persons and the costs for loss of work productivity, for example, days lost at work, but also the loss of work productivity for accompanying persons. Other indirect costs derive from the consequences of an advanced disease stage, for example, inability to drive, increased risk of falls, and depression triggered by the disease, for example. Another aspect, the quality of life of a patient, is also seldom addressed in studies. A European study [7] showed that the costs of glaucoma care have a significant linear trend parallel to the increasing severity of the disease. It is important to recognize that glaucoma is a chronic disease, which progresses in every patient. It is crucial to differentiate between fast progressors and slow progressors to keep costs under control [8] because the frequency of outpatient visits can vary between fast and slow progressors.

Looking at the studies on direct costs of glaucoma care in different countries, the main message was that the aspect of costs should be discussed with the patient especially in regard to the price of antiglaucomatous drugs, which are quite expensive in some countries (e.g., the United States). Costs for antiglaucomatous drugs must be judged in comparison to the median income and not as a total amount. An important study concerning the aspect of costs came from Nigeria, where the costs for glaucoma drugs was 50% of the monthly income of a middle-class family, but 100% of a lower-class family [9].

In the recent years, generic medications have taken over the antiglaucomatous drug market. The price of a generic drug is much lower compared to the branded drug, but many studies [10,11] have shown that they are not equal: only the main substance needs to be identical to the branded drug. The remaining, especially the preservative agent, but also the consistency of the container and the size of the drop can differ. In addition, the concentration of the main substance is allowed to vary within certain limits as well. Switching from a branded drug to a generic drug means introducing a new medication with more costs in regard to follow-up visits and to informing the patients about this topic. It is crucial to make ophthalmologist and pharmacists aware about the differences between branded and generic drugs. Interestingly, a large difference in the prescription habits of generic drugs exists between different countries. In Europe, the northern countries prescribe more generic drugs than the southern countries [12].

### 3.2. Patient's Adherence

Adherence or the synonym compliance is defined as the cooperation of the patient with the recommendation given by the treating doctor. The term persistence on the other hand describes the length of time the patient uses the medication as prescribed [13].

Glaucoma, as other chronic diseases, has a low adherence and persistence rate. Many patients, especially in the early phases of their disease, do not realize the consequences of progression. The so-called “white coat adherence” is seen frequently: the patient applies the drops only a few days before he or she visits his or her doctor and stops to take them shortly after the visit again. Many studies have discussed the theme adherence, but as Cate [14] pointed out in 2015, there are inconsistencies among different monitoring strategies and adherence measures. Common obstacles to adherence can be grouped into four categories: situational and environmental factors; medication regimens; patient's factors; and doctor's factors [15]. Newman-Casey [16] mentioned among the most often cited factors for nonadherence psychological factors (for example, low self-efficacy and forgetfulness), difficulty with drug administration (especially in patients with rheumatic diseases or in patients with dementia), and medication scheme. Hasebe [17] and Movahedinejad [18] found similar reasons and added that an important factor was the lack of awareness regarding the complications of progressive glaucoma. A practical comment was published by Muir [19], who said that adherence involves four steps.

The patient needs to get the medication, he or she has to be physically able to apply the drop in the eye, and use the medication at the appropriate time. Lastly, he or she needs to repeat these three steps every day.

Interestingly, the patient's declaration about their adherence often differs with the rates actually measured. Gatwood [20] found a great discrepancy between the patient reported data and the actual measurements obtained by a wireless device.

Having access to electronic information should improve adherence; hence, Newman-Casey [21] and Fiscella [22] showed in studies that neither the availability of information sent via mail nor access to electronic information improved adherence.

An interesting study by Rees [23] looked at cultural differences to adherence. They found that in Western cultures the beliefs about glaucoma treatment were predictive of adherence.

It is well known that glaucoma treatment often leads to local and systemic side effects. Zimmermann [24] found in a study that up to two-thirds of glaucoma patients suffer from side effects. The side effects derive either from the medication itself or from the preserving agents of the drops. It is mandatory to find an adequate therapy and perhaps change from a preservative containing to a preservative free medication, which might improve the ocular surface and lead to less local side effects.

A special interest has been given lately to the glaucoma management of elderly patients. Different factors influence the behavior of an elderly patient: often, they take other medication to treat systemic diseases and the introduction of an antiglaucomatous drug needs to be discussed with the family doctor. Other influencing factors are rheumatic diseases or a possible dementia, interfering with reliable application of drugs.

In an elderly patient, other treatment options than local drugs must be discussed: a good alternative are laser treatments or surgical options [25].

We tend to undertreat elderly glaucoma patients because the lifespan is much longer than in earlier years and patients might realize progressive visual field defects, as seen in an increased tendency for falls and a reduced capacity for driving a car [25].

How can we improve the adherence of our patients?

To improve adherence, different factors need to be addressed: the medication itself, local factors, the application of the medication, the systemic factors, and last but not least the costs.

Many antiglaucomatous drugs lead to local side effects and may be exchanged by another drug of the same class with less side effects. Leung [26] and Fechtner [27] showed in studies that about 50% of patients using local antiglaucomatous medication suffered of more or less severe signs and symptoms of ocular surface disease (OSD) and eventually had to stop the medication.

Applying the drops the correct way is challenging. Patients with rheumatic disease are often not able to open drug containing bottles or single use units [28].

It is important to show the patient how to apply the drops correctly. Atey [29] showed in a study that an improvement of instillation of eye drops leads to a reduction of IOP. Some patient may misunderstand the instruction of the doctor: twice a day might mean at 8 am and at 8 pm, but patients may interpret the instruction another way and apply the drops in the morning and at lunch.

The systemic problems were addressed above: often, the patients with glaucoma have many other drugs prescribed by their general practitioner and it is important to talk to the general practitioner before introducing another topical medication.

The cost issue is an important factor and can lead to a reduced adherence if the patient cannot afford the medication prescribed [30].

As it is well known that glaucoma has a low adherence and persistence rate, much effort is given to ameliorate and simplify the administration of drugs: drug combinations replace the addition of bottles and longer-lasting products, for example, in the form of slow release drugs or intraocular injections, are studied.

### 3.3. Laser and Minimally Invasive Glaucoma Surgery to Improve Adherence

Multiple studies on laser trabeculoplasty and minimally invasive glaucoma surgery (MIGS) procedures have been published in the recent years. Recently, the LIGHT study got published [31]. The LIGHT trial was a randomized controlled trial with 36 months of follow-up comparing selective laser trabeculoplasty (*n* = 356) to drops (*n* = 362) in treatment-naïve patients. Eyes of patients in the selective laser trabeculoplasty group were within target intraocular pressure at more visits (93·0%) than in the eye drops group (91·3%) [31]. In addition, selective laser trabeculoplasty was more cost-effective in the United Kingdom compared to drops. The increasing interest in MIGS procedures is based on their favorable risk profile and at least moderate efficacy, which makes these procedures useful for moderate and early stages of glaucoma with mal-compliance or intolerability to topical therapy. The majority of MIGS procedures enhance conventional/trabecular outflow. Thus, a target pressure cannot be expected to be below 14–16 mmHg. In addition, MIGS procedures like the XEN gel stent (Allergan Inc.) or the PRESERFLO MicroShunt (Santen Inc.) bypass conventional and alternative outflow pathways and guide aqueous humor from the anterior chamber through the implant into the subconjunctival space. Generally, these procedures lower IOP independently from the patient's adherence to topical medications. Because the IOP lowering efficacy of a MIGS procedure is not dependent on the patient's adherence to the prescribed drops, they should logically enhance treatment success compared to topical therapy which on the other hand is dependent on the patient's adherence.

### 3.4. Does a Connection between Costs and Adherence Exist?

Not many studies looked at the connection between costs and adherence. Disease progression and severity of the disease are probably the most relevant factors for adherence. However, costs of glaucoma medication, which increase in patients with nonadherence, should not be underestimated. As we mentioned above, Traverso discussed in a European study that the costs increased linearly with the severity of the disease [7]. A connection between costs and adherence exists probably via the progression of the disease: in a progressing disease, the costs are rising [7, 30]. However, more studies are needed to determine the influence of adherence on glaucoma progression and, thus, on the costs of glaucoma care. It is the duty of ophthalmologists, however, to improve patient's adherence to prescribed therapies [30].

## 4. Summary

Adherence has a major input on the outpatient care of glaucoma patients. In glaucoma, as in other chronic diseases, adherence is rather low. Interestingly, the adherence rate has not improved over the last decades despite better information of the patient about their disease and improvement in medical and surgical therapies [32]. Low adherence may lead to progression of the disease and therefore to higher costs. More studies are needed to evaluate the influence of low adherence on progression of the disease and to calculate the costs deriving from progression.

However, the duty of the treating ophthalmologist is to improve patient's adherence, mainly by informing the patient and by finding an adequate glaucoma treatment, which fits into the patient's lifestyle.
